# Inhibitors of the FASII Metabolic Pathway in *Toxoplasma gondii*: Advances and Therapeutic Perspectives

**DOI:** 10.3390/microorganisms14051072

**Published:** 2026-05-09

**Authors:** Claudia Jessica Castillo-Villanueva, Jhony Anacleto-Santos, Teresa de Jesús López-Pérez, Brenda Casarrubias-Tabarez, Teresa I. Fortoul, Marcela Rojas-Lemus, Nelly López-Valdez, Elisa Vega-Ávila, Fernando Calzada, Perla Yolanda López-Camacho, Norma Rivera-Fernández

**Affiliations:** 1 Posgrado en Ciencias Biológicas, Ciudad Universitaria, Universidad Nacional Autónoma de México (UNAM), Ciudad de México 04510, Mexico; jessicacastillo@unam.edu; 2 Departamento de Microbiología y Parasitología, Facultad de Medicina, Ciudad Universitaria, Universidad Nacional Autónoma de México (UNAM), Ciudad de México 04510, Mexico; jhony@unam.mx (J.A.-S.); tere.lopez82@comunidad.unam.mx (T.d.J.L.-P.); 3 Departamento de Biología Celular y Tisular, Facultad de Medicina, Ciudad Universitaria, Universidad Nacional Autónoma de México (UNAM), Ciudad de México 04510, Mexico; bcasarrubias@facmed.unam.mx (B.C.-T.); fortoul@unam.mx (T.I.F.); marcelarojas@comunidad.unam.mx (M.R.-L.); nellylopez@facmed.unam.mx (N.L.-V.); 4 Departamento de Ciencias de la Salud, Universidad Autónoma Metropolitana Iztapalapa, Ciudad de México 09340, Mexico; vega@xanum.uam.mx; 5 Unidad de Investigación Médica en Farmacología, Unidad Médica de Alta Especialidad, Hospital de Especialidades Centro Médico Nacional Siglo XXI, Instituto Mexicano del Seguro Social, Col. Doctores, Cuauhtemoc 06725, Mexico; fernando.calzada@imss.gob.mx; 6 Departamento de Ciencias Naturales, Unidad Cuajimalpa, Universidad Autónoma Metropolitana (UAM), Col. Santa Fe, Cuajimalpa, Ciudad de México 05348, Mexico; plopezc@cua.uam.mx

**Keywords:** *Toxoplasma gondii*, *anti-Toxoplasma* compounds, lipidome, apicoplast, FASII, lipid inhibitors, fatty acid metabolism

## Abstract

*Toxoplasma gondii* is an obligate intracellular parasite of the phylum Apicomplexa and the causative agent of toxoplasmosis, a disease with a worldwide distribution that causes serious consequences in immunocompromised patients and during pregnancy. Current pharmacological treatments have significant limitations, including their toxicity, lack of efficacy against the chronic phase of the parasite, and low selectivity, highlighting the need to develop new therapeutic targets. One of the most promising targets is the fatty acid synthesis pathway II (FASII), a metabolic pathway located in the parasite’s apicoplast and absent in mammalian hosts. This review synthesizes the available evidence on FASII pathway inhibitors described to date, as well as their potential impact on the viability and development of *T. gondii*. Overall, the reviewed studies support the FASII pathway as an attractive therapeutic target for the development of more selective and effective treatments against toxoplasmosis.

## 1. Introduction

*Toxoplasma gondii* is an obligate intracellular protozoan of the phylum Apicomplexa, capable of infecting a wide variety of homeothermic animals, including humans, in whom it can cause an infection known as toxoplasmosis. It is considered one of the most biologically successful parasites in its group, with a global seroprevalence of 31% [1,2]. However, these figures can vary from 10% to over 90% depending on the region. In general, coastal areas tend to have higher rates, and these differences are related to factors such as climatic conditions (high temperatures and humidity) and the dietary and hygiene habits of each population [3,4,5].

*Toxoplasma gondii* is transmitted primarily through the ingestion of food or water contaminated with oocysts or by consuming raw or undercooked meat containing tissue cysts, although it can also be transmitted vertically or through blood transfusions and organ transplants [6]. While the infection is usually asymptomatic in immunocompetent individuals, it poses a significant clinical risk for immunocompromised persons, especially patients with HIV/AIDS, in whom reactivation can lead to toxoplasmic encephalitis, and for pregnant women, in whom it can cause spontaneous abortion or congenital toxoplasmosis [7,8]. Additionally, chronic infection has recently been associated with behavioural alterations and psychiatric disorders [9,10].

Currently, there are no vaccines available for human use to treat the infection; therefore, treatment is based on pharmacological therapy, with the combination of pyrimethamine and sulfadiazine (P/S) being the drugs of choice for the various clinical manifestations, including acute toxoplasmosis, toxoplasmic encephalitis, and ocular toxoplasmosis [11,12]. Despite its efficacy against tachyzoites, the P/S combination is ineffective against bradyzoites, the parasitic form responsible for chronic toxoplasmosis and reactivation of the acute phase in immunocompromised patients. P/S inhibits the enzymes involved in folic acid metabolism; however, the treatment does not discriminate between the parasite’s and the host’s enzymes, leading to hematological toxicity characterized by megaloblastic anemia, leukopenia, and thrombocytopenia, as well as hypersensitivity reactions ranging from mild rashes to severe manifestations such as Stevens-Johnson syndrome or toxic epidermal necrolysis [11,13]. Macrolides (azithromycin and spiramycin) and lincosamides (clindamycin) inhibit the 50S ribosomal subunit, altering protein synthesis in the parasite’s apicoplast [14,15]. However, these drugs are also ineffective against acute toxoplasmosis and produce mild side effects [16,17].

Spiramycin is used in pregnant women during the first trimester to reduce the risk of mother-to-fetus transmission, although its low placental penetration makes it ineffective against an already established fetal infection [18]. Given the global prevalence of *T. gondii* and the severity of the disease in vulnerable populations, it is important to develop new anti-Toxoplasma compounds that are less toxic, effective against all parasitic stages, and exhibit high selectivity [2].

## 2. Lytic Cycle of *Toxoplasma gondii*

**Figure 1:**
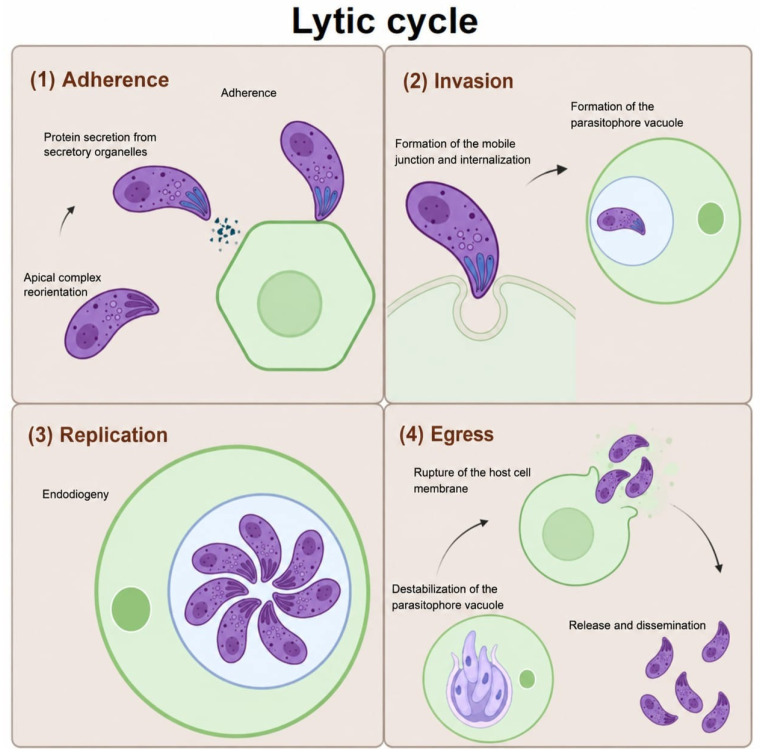
Lytic cycle of *Toxoplasma gondii*. The cycle compromises four main stages: (**1**) adhesion, in which the tachyzoite recognizes and binds to the host cell via proteins secret by the micronemes; (**2**) invasion, characterized by the formation of the sliding junction and the establishment of the parasitophorous vacuole; (**3**) replication, during which tachyzoites divide by endodyogeny within the parasitophorous vacuole; and (**4**) egress, which involves the rupture of the vacuole and the host cell membrane, allowing the release of parasites to restart the infectious cycle. Diagram created with BioRender.com by Castillo CJ. under licence to publish.

The lytic cycle is the process during which tachyzoites attach to and invade a host cell, establish themselves inside it by forming a parasitophorous vacuole, replicate by endodyogeny, and exit, causing cell lysis and releasing new parasites that can invade more cells (Figure 1). Since this process is similar to the way viruses replicate, it is called the lytic cycle of *T. gondii* [19].

The lytic cycle depends mainly on three specialized secretory organelles: micronemes, rhoptries, and dense granules. These organelles, located mainly at the apical end of the parasite, sequentially release a series of effector proteins that allow the parasite to carry out its intracellular cycle. Micronemes release MIC proteins that allow the tachyzoite to slide and adhere to the cell surface and initiate the invasion process [20]. Subsequently, rhoptries discharge their RON and ROP proteins to form a mobile junction and build the parasitophorous vacuole. Finally, dense granules secreted GRA proteins that modify the intracellular environment and the parasitophorous vacuole, promoting parasite replication and participating in egress [20,21].

The host’s immune response induces changes in gene expression, translation, and metabolism in tachyzoites, which halt their rapid and active division, giving way to slow replication and conversion into bradyzoites that will form tissue cysts from host cells. Tissue cysts can remain in tissues such as skeletal muscle and the brain throughout the host’s lifetime [20,22].

In immunocompromised patients, bradyzoites can reactivate and differentiate into tachyzoites, causing severe or potentially fatal acute disseminated infection [22]. The replication of the parasite through the lytic cycle requires intense membrane remodelling and an increase in the synthesis of lipids from pathways such as FASII, essential for the rapid formation of parasitophorous vacuoles and systemic dissemination [22].

## 3. *Toxoplasma gondii* Lipidome

The lipidome of *T. gondii* is a set of lipids that the parasite produces, uses, or modifies and plays a fundamental role in all stages of the lytic cycle, from invasion to exit. Lipids act as important components of cell membranes, organelles, and as signalling molecules in intracellular communication [19,23].

**Figure 2:**
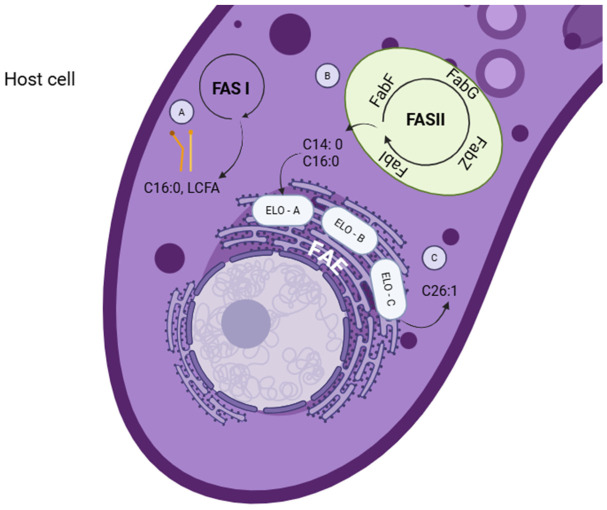
Fatty acid synthesis pathways in *Toxoplasma gondii*. (A) FAS I pathway: cytosolic fatty acid synthesis mediated by a type I multifunctional synthase. (B) FAS II pathway: de novo synthesis in the apicoplast via type II enzyme system. (C) Elongation pathway (ELO): extension of preformed acyl chains in the endoplasmic reticulum. Figure created using BioRender.com under licence to publish by Castillo CJ. under licence to publish.

*Toxoplasma gondii* obtains fatty acids de novo through three pathways (Figure 2): type I fatty acid synthesis (FASI), which generates palmitic acid and long-chain fatty acids in the cytoplasm, FASII that generates short-chain fatty acids such as myristic and palmitic acids in the apicoplast, and fatty acid elongation that generates long-chain fatty acids such as palmitoleic and oleic acids in the endoplasmic reticulum [24,25]. This pathway is unique to the parasite and is considered an important metabolic target for the development of new drugs against toxoplasmosis [26]. *T. gondii* lipid inhibitors have the ability to directly affect various lipid components, destroy membranes, and ultimately kill the parasite [26,27].

## 4. FASII Synthesis Pathway

The metabolic pathway known as FASII is present in some bacteria, plants, and parasites, including those belonging to the phylum Apicomplexa. This pathway functions through the sequential action of individual monofunctional enzymes to generate fatty acids [28]. Unlike the FASI pathway found in mammals, the FASII pathway allows for greater flexibility in the length and composition of the synthesized fatty acids. The authors have suggested this metabolic pathway as a highly promising therapeutic target for the creation of new compounds with antiparasitic and antibacterial activity because it is absent in humans [27,28].

**Figure 3:**
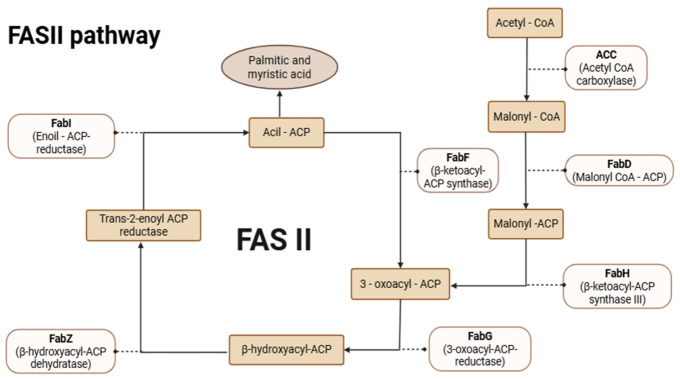
Schematic representation of FASII in *Toxoplasma gondii*. The dotted arrows indicate the involvement of the enzymes that catalyze each step of the pathway. Figure created with BioRender.com by Castillo CJ. under licence to publish.

The FASI and FASII pathways have both structural and functional differences; FASI consists of a single, large protein complex containing multiple catalytic sites, while FASII functions as a system composed of individual enzymes that act in a coordinated manner through a series of cyclic and sequential elongation reactions [28,29]. During each cycle, the growing acyl chain remains covalently bound to the acyl carrier protein (ACP) along the pathway and these reactions are catalyzed by the enzymes β-ketoacyl-ACP synthase, β-ketoacyl-ACP reductase, β-hydroxyacyl-ACP dehydratase, and enoyl-ACP reductase (ENR) (Figure 3) [29,30].

The parasite obtains glucose from the cytoplasm through the action of pyruvate dehydrogenase (PDH) to form the initial substrate, acetyl-CoA, and ultimately produces short-chain saturated fatty acids, such as myristate (C14:0) and palmitate (C16:0) (Figure 3) [31]. Another function of FASII is the production of octanoic acid (ACP), a precursor of lipoic acid. Lipoic acid is a necessary cofactor for the activity of the E2 subunit of apicoplastic pyruvate dehydrogenase (PDH), which is also important for the supply of acetyl-CoA [32,33].

## 5. The FASII Pathway as a Drug Target

Given concerns surrounding drug resistance and the limitations of current treatments for toxoplasmosis, the FASII pathway represents a very interesting pharmacological target [34].

The differences between the FASII pathway found in the parasite and the eukaryotic FASI pathway in the host make it possible to design selective drugs that can reduce side effects in humans (Table 1) [34].

*Toxoplasma gondii* can only obtain some of its lipids directly from the host cell. Therefore, the FASII pathway is essential for obtaining fatty acids that the host cannot provide, such as myristic and palmitic acids [26,35]. Authors mention that the FASII pathway is necessary for the parasite’s growth, survival, and pathogenesis, thus compromising its virulence [36]. Alterations in this pathway have also been reported to cause defects in cytokinesis and incomplete pellicle formation in the parasite’s tachyzoites, affecting their proliferation [36,37].

Genetic research on the loss of lipid metabolism function in the apicoplast demonstrates the importance of the FASII pathway for *T. gondii*. For example, alterations in the endoplasmic reticulum acetyl-CoA transporter (TgAT1) and a lack of precursors affect de novo fatty acid synthesis in the apicoplast [38]. This leads to organelle formation abnormalities and a significant reduction in parasite proliferation in vitro. Furthermore, the lack of pyruvate supply to the apicoplast, which is necessary for lipoylation and apicoplast PDH activity, also causes organelle loss and decreased parasite growth, justifying the use of FASII as a therapeutic target [29,38].

## 6. FASII Inhibitors

### 6.1. Triclosan

Triclosan is a diphenyl ether widely used in household products; its structure is that of a 2-hydroxy diphenyl ether, and it was one of the first compounds identified to act on bacterial FASII. Due to its broad-spectrum antibacterial activity, triclosan is used in soaps, deodorants, hand lotions, toothpastes, and impregnated plastics [39,40].

Triclosan (5-chloro-2-[2,4 dichlorophenoxy] phenol) was shown to inhibit the growth and survival of the Apicomplexa parasites *Plasmodium falciparum* and *Toxoplasma gondii* by inhibiting the enzyme enoyl-ACP reductase (ENR or FabI) (TgENR in *T. gondii)* [39,41].

FabI is the enzyme responsible for the final reduction in the FASII pathway, catalyzing the NAD+-dependent reduction of a trans-2,3-enoyl portion to a saturated acyl chain [41]. This enzyme has been described as an ideal pharmacological target because it has no homologs in mammals [41,42].

Triclosan has been reported to affect parasite growth at nanomolar (nM) concentrations. The median inhibitory concentration (IC_50_) described in vitro for inhibiting the growth of tachyzoites of the RH strain is approximately 200 nM [41,42].

In an in vitro enzyme assay, triclosan has been shown to inhibit the enzymatic activity of TgENR at IC_50_ values below 20 nM with a specific IC_50_ value of 15 nM [41]. Alterations in apicoplast morphology and reduced proliferation were observed when the enzyme’s catalytic activity decreased [43,44]. Reports on the in vivo effects of triclosan indicate that it is a potential drug against murine toxoplasmosis, in both its acute and chronic forms [45,46].

Furthermore, some authors have demonstrated that triclosan significantly reduces mortality in mice and the parasite load in the peritoneum and liver. It also decreases the viability and virulence of tachyzoites and cysts from infected and treated mice [46,47]. Triclosan acts as a non-competitive inhibitor of the NAD+ cofactor, forming a ternary complex with the enzyme and the oxidized form of the cofactor: triclosan/NAD+/TgENR [32].

Inhibition occurs through the binding of triclosan to a conserved tyrosine (Tyr) residue in the active site of the enzyme, which prevents the formation of a ternary complex with the substrate [42]. Structurally, the tight binding involves the formation of an alpha helix over the inhibitor binding site, a feature that has been observed in the crystal structure of TgENR co-crystallized with triclosan and NAD+ [48].

The phenolic ring (ring A) of triclosan is positioned in a sandwich configuration opposite the NAD+ cofactor, facilitating ***π***-stacking interactions [49]. The phenolic hydroxyl group of ring A participates in hydrogen bonds with the conserved residue Tyr189 and with the 2′ hydroxyl group of NAD+ [49,50]. The dichlorophenoxy ring (ring B) is mainly involved in Van der Waals interactions within a pocket bounded by several residues, including Leu128, Ala131, Val134, Met193, Ala231, and Ile235; although this binding is reversible, its tight bond and very slow dissociation rate contribute to its efficacy [42,51].

Despite its potential enzymatic inhibitory activity, some limitations have been reported for the systemic therapeutic use of triclosan. For example, triclosan has been reported to have low water solubility, a high ClogP value, and low bioavailability [52]. On the other hand, toxicity has been reported, as triclosan impairs muscle contractility [53].

Due to these limitations, triclosan has been used as a scaffold for the design of new compounds with the aim of improving their ADMET (absorption, distribution, metabolism, excretion, and toxicity) profiles [42].

Modification of the A and B rings of triclosan can optimize ADMET profiles by increasing drug permeability; therefore, analogues with substitutions at positions 4′, 5′, and 6′ of triclosan have been developed [32].

One strategy to mitigate adverse effects and low solubility has been to incorporate triclosan into the lipid bilayer of liposomal nanoparticles. This formulation improved its efficacy, allowing its use at lower doses and reducing adverse biochemical effects [45,47].

Triclosan is an important starting point in the research of drugs against toxoplasmosis due to its potent inhibition of the essential enzyme TgENR, although its unfavourable physicochemical properties have prompted research into analogues [32].

### 6.2. Triclosan-Derived Inhibitors

Inhibitors based on the triclosan skeleton have been developed, which, as mentioned above, is known for its ability to inhibit the FabI enzyme. However, this compound has significant limitations, such as low bioavailability and toxicity issues. For this reason, numerous triclosan analogues, mainly diaryl ethers, have been designed and evaluated to optimize their physicochemical properties, such as absorption, distribution, metabolism, excretion, and toxicity (ADMET), and to increase their permeability to the parasite’s apicoplast [54].

The inhibition of TgENR by triclosan is based on the formation of a slow, tight-binding ternary complex with the enzyme and the cofactor in its oxidized form (NAD+) [32]. This mechanism involves π-stacking interactions between the phenolic ring (A) of triclosan and NAD+, as well as hydrogen bonds between the hydroxyl group of triclosan, the ether bond and a conserved tyrosine residue, and the 2′-hydroxyl of NAD+ [49]. All potent analogues share this same mechanism of action, binding exclusively to the TgENR/NAD+ complex [32].

Tipparaju et al. (2010) [54] reported 53 new compounds, all inhibitors of the enzyme enoyl ACP reductase (TgENR); the analogues that have been developed are presented below, grouped according to the modified position they present [54].

### 6.3. Analogues with Substitutions in Ring B (Position 4′)

These analogues focused on modifying the 4′ position of ring B (dichlorobenzene) to improve ADMET properties and enhance substrate entry [49,50].

### 6.4. Triazole Analogues (Series **16a**, **16b**, **16c**)

Compound **16c** (1-[3-chloro-4-(4-chloro-2-methoxyphenoxy) benzyl]-1H-1,2,3-triazole): Features a chemical modification in the unsubstituted triazole group at the 4′ position of ring B [50]. This compound binds in a manner similar to triclosan, preserving crucial interactions. The triazole structure allows the formation of two new hydrogen bonds with the nitrogen atoms at positions 2 and 3 of the triazole ring and the NH groups of Asn130 and Gly131 of TgENR [50].

An MIC_50_ = 0.25 µM is reported, which is approximately four times better than triclosan. The IC_50_ = 26 nM obtained is very similar to that reported for triclosan; in addition, its physicochemical properties evaluated were favourable in terms of ClogP, water solubility (Sw), and permeability in Caco-2 cells [50].

Reports of its in vivo efficacy show that the compound did not reduce the parasite load at 10 mg/kg, which is the effective dose for triclosan, but it protected mice by reducing the parasite load at a higher dose of 75 mg/kg. It is 10 times less toxic than triclosan in mice [50].

Compound **16a** (2-[4-[(4-Butyl-1H-1,2,3-triazol-1-yl) methyl]-2-chlorophenoxy]-5-chlorophenol): It has a chemical modification, the 4-butyltriazole group at the 4′ position of ring B. It binds in a very similar way to 16c, but the n-butyl chain introduces steric hindrance, which affects the alignment of ring A in the active site, weakening the π-stacking interactions with NAD+ and resulting in an IC_50_ slightly higher than that of 16c [50]. Stec et al. [50] report that the compound has high antiparasitic activity, as its IC_50_ was determined to be 43 nM; however, its ADMET properties were less favourable than those of compound 16c [50].

Compound **16b** (5-Chloro-2-[2-chloro-4-[(4-phenyl-1H-1,2,3-triazol-1-yl)methyl] phenoxy]phenol): It has a modification in the 4-phenyltriazole group at the 4′ position of ring B. It is reported that the 4-phenyltriazole group generated effects on the binding mode, since the hydrogen bonds with Asn130 and Gly131 observed with compound 16c are not formed, which decreases the activity of the inhibitor. An MIC_50_ = 1 µM and moderate enzyme inhibition of 82% at 1 µM were determined [50].

Compound **41a** (5-Chloro-2-[2-chloro-4-[(hexylamino)methyl] phenoxy]phenol): It has a substituent at the 4′ position; it is suggested that its parasitic activity could be due to interaction with targets other than TgENR. A MIC_50_ value of 1 µM is reported, and it is an enzymatically inactive compound, since only 25% inhibition of parasitic activity was observed at 1 µM [50].

Compound **41b** (5-Chloro-2-[2-chloro-4-[(phenylamino)methyl] phenoxy]phenol): It has an aniline substituent at the 4′ position; the biaryether portion fits well into the substrate binding site, almost identical to triclosan, which explains its high enzymatic activity. Its IC_50_ is 31 nM, and 94% of enzymatic activity is reported at 1 µM [50].

Compound **37a** (N-[3-Chloro-4-(4-chloro-2-hydroxyphenoxy) benzyl]-5-methyl-1,2-oxazole-3-carboxamide): It has a 5-methylisoxazole group attached by a carboxamide ligand at 4′; it has been reported that this binds similarly to triclosan through additional stabilization by hydrogen bonds between the amide oxygen of the compound and the amide NH of Asn130 and Gly131 [50,55]. It has an MIC_50_ = 5 µM and an IC_50_ = 100 nM, with enzyme inhibition of 88% at 1 µM [50].

Compound **37c** (N-[3-Chloro-4-(4-chloro-2-hydroxyphenoxy) benzyl]-2-(3-methyl-1,2-oxazol-5-yl) acetamide): It has a (3-methyl-isoxazo-5-yl)-acetamide group at 4′; it is reported to bind similarly to triclosan through stabilization by hydrogen bonds between the amide oxygen and the amide NH of Asn130 and Gly131 [55]. An IC_50_ = 33 nM and 96% inhibition at 1 μM were obtained. In vivo studies report similar efficacy to compound 16c; it was not effective at 10 mg/kg, but protected mice by reducing the parasite load at 75 mg/kg [50,56].

Compounds **24a** and **24b** (2-[4-(5-Butyl-1,2-oxazol-3-yl)-2-chlorophenoxy]-5-chlorophenol and 5-Chloro-2-[2-chloro-4-(5-phenyl-1,2-oxazol-3-yl) phenoxy] phenol): This is modified with oxazole groups substituted with butyl in compound **24a** and with phenyl at 4′ in compound **24b**. Stec et al. [50] report an IC_50_ of 18 nM for compound **24a** and 28 nM for **24b**; these compounds are reported to have good affinity for the active site of the enzyme [50].

### 6.5. Analogues with Substitutions in Ring A (Position 5)

The modifications in position 5 (phenolic ring) are based on the structural difference between TgENR, which has a conserved alanine residue, and its bacterial and plant homologues, creating more space to introduce substituents [49,50].

Compound **33** (N-[4-(2,4-Dichlorophenoxy)-3-hydroxybenzyl]-5-methyl-1,2-oxazole-3-carboxamide): This has a 5-methylisoxazole group at position 5′; molecular docking suggested that the compound initially interacts in a reverse binding mode, where the isoxazole ring interacts via π-stacking with NAD+, and the amide oxygen forms H bonds with the 2′ hydroxyl of NAD+. The phenolic ring A is positioned almost perpendicular. However, it is postulated that the plasticity of the ENR active site could accommodate ring A without the need for an inverted mode [50]. Very high enzymatic activity is reported, as its IC_50_ = 19 nM and the inhibition of enzymatic activity at 1 μM is 96%, and its ADMET characteristics are favourable [50].

Compounds **9a**, **9b**, and **9c** (5-[(4-Butyl-1H-1,2,3-triazol-1-yl) methyl]-2-(2,4-dichlorophenoxy) phenol, 2-(2,4-Dichlorophenoxy)-5-[(4-phenyl-1H-1,2,3-triazol-1-yl) methyl] phenol and 2-(2,4-Dichlorophenoxy)-5-(1H-1,2,3-triazol-1-ylmethyl) phenol: These are triazole analogues with modification at position 5 (**9a**: 4-butyltriazole; **9b**: 4-phenyltriazole; **9c**: triazole). Their binding mechanism is different from that reported for triclosan; it is determined by the triazole group that binds to the entry portal, forming H-bonds with Asn130 and Gly131. Ring A is displaced perpendicularly. The lack of parasitic activity is attributed to its predicted low solubility and restricted access to the apicoplast [50].

Compound **39b** (5-[(Cyclohexylamino)methyl]-2-(2,4-dichlorophenoxy) phenol): With a cyclohexylamine substituent at position 5, its parasitic activity is likely the result of interaction with targets other than TgENR. In addition, it showed excellent activity against *P. falciparum* (IC_50_ = 0.03 μM), reinforcing the hypothesis of a target other than ENR in *T. gondii,* since 34% enzyme inhibition is reported at 1 μM [50].

### 6.6. Analogues with Substitutions or Complete Replacement of Ring B

These analogues were designed primarily to improve solubility by exploiting the channel leading from the triclosan binding site to the external solvent (ACP entry portal) [42].

Compound **5c**: This compound replaces the chlorine at C4′ with an N-acetylated piperazine moiety. It is suggested that the compound forms a hydrogen bond between the N4 of the piperazine ring and the oxygen atom of the side chain of the conserved residue Asn130. These modifications improved solubility, reporting a value of 160 mg/L, which is higher than that reported for triclosan at 4.6 mg/L; the inhibition of TgENR enzyme activity at 1 μM was 81%, and the authors reported low toxicity in host cells [42,50].

Compound **14b**: This compound replaces ring B with a furan ring substituted with an amide/isoxazole group at C5; molecular docking suggests that Asn130 could play an important role in binding by forming a hydrogen bond with the inhibitor. The compound has good solubility (210 mg/L) and 72% enzyme inhibition at 1 μM with low toxicity to host cells [42].

Compound **18**: Replacement of ring B with a thiophene ring linked to a 4-acetylpiperazin-1-yl group via a methylene spacer; the binding mechanism is based on the flexible methylene that allows the piperazine ring to adopt a favourable position to form a hydrogen bond with Asn130. The authors report that this compound exhibited one of the best solubilities, with a solubility value of 2200 mg/L and 73% inhibition of enzyme activity at 1 μM, in addition to low toxicity [42].

### 6.7. Potent Inhibitors

A thermal displacement assay (TSA) was used to differentiate the affinity of potent inhibitors with IC_50_ in the low nanomolar range that could not be differentiated with standard enzyme activity assays. All compounds tested using this technique (20 in total) shared the same mechanism of action as triclosan, i.e., forming a ternary complex with TgENR and NAD+ [32,57,58,59].

Six compounds showed Kd (dissociation constant) values in the low femtomolar (fM) range when measured at high NAD+ concentration (6 mM), indicating an affinity similar to or greater than that of triclosan [32,42].

Four of the compounds analyzed (**5**, **9**, **10**, and **15**) inhibited the growth of *T. gondii* parasites with a potency equal to or greater than that of triclosan. Compound **5** has small substituents at the 4′ position, compounds **9** and **10** have a benzylamino portion, and compound **15** has a modification at the 6′ position [32].

### 6.8. Inactive Analogues

Compounds with substitutions at 2′: Analogues modified at the 2′ position of triclosan did not show significant inhibitory activity against the TgENR enzyme due to probable steric clashes with the NAD+ cofactor or the binding site [32,42].

Ketone analogues **43a** (1-[4-(2,4-Dichlorophenoxy)-3-hydroxyphenyl] propan-1-one) and 43b (4-(2,4-Dichlorophenoxy)-3-hydroxyphenylmethanone): They showed only weak enzymatic inhibition activity and were ineffective in inhibiting complete cell growth of the parasite [32,42].

Amide analogues **30a-d** (4-(2,4-Dichlorophenoxy)-3-hydroxy-N-phenylbenzamide; 4-(2,4-Dichlorophenoxy)-3-hydroxy-N, N-dibutylbenzamide; 4-(2,4-Dichlorophenoxy)-3-hydroxyphenylmethanone; 4-(2,4-Dichlorophenoxy)-N-hexyl-3-hydroxybenzamide): They showed no significant activity in any of the biological assays, even though modelling predicted reasonable binding [32,42]. Triclosan, due to its limitations related to physicochemical properties, enzymatic challenges, absorption, and toxicity, has hindered the development of safe and efficient drugs; however, the compound serves as a base scaffold for the development of all analogues that seek to address the aforementioned limitations [54,60].

Although the analogues were designed to improve ADMET properties, several deficiencies persist, such as the lack of correlation between enzymatic and parasitic activity in the in vitro models, as many analogues were shown to have very high enzymatic activity but low or even no parasitic activity [42]. The lack of parasitic activity is attributed to their predicted low solubility and restricted access to the parasite’s apicoplast [42]. It is important to mention that analogues must be able to reach the apicoplast, where the target enzyme is located, which requires the drug to overcome multiple barriers (host cell membrane, parasite membrane, and plastid membrane) [32].

Promising analogues that were tested in vivo showed limited efficacy; the cause of this low potency in vivo is associated with the high rate of hepatic metabolism, with a short half-life (29 min in cryopreserved human hepatocytes), which explains the need for a much higher dose to observe a protective effect [50].

### 6.9. Thiolactomycin

Thiolactomycin (TLM) has a unique γ-thiolactone ring and is a promising candidate as an antibiotic that acts specifically on bacterial type II fatty acid synthesis [61]. This compound has been investigated for its antimicrobial potential and has been shown to have activity against the in vitro growth of the parasite. It is considered an inhibitor of the FASII pathway because the mechanism of action of TLM targets the enzyme α-ketoacyl-ACP synthase (Fab H), which catalyzes a step in the FASII pathway [30,41].

Analogues of the compound have been studied as a strategy to optimize some of the pharmacological properties of the initial compound, which is an approach that is also applied to other potent inhibitors with limitations [32,42]. Similarly, to TLM, each of the analogues is designed to target the FASII pathway of *T. gondii* [46]. Eight new TLM analogue compounds have been evaluated in vitro in the RH strain of *T. gondii* (using LLC-MK2 cells); the activity of these analogues was measured by IC_50_ determination, lipid extraction, and chromatographic analysis [62,63]. Mean IC_50_ values ranging from 1.6 to 29.4 µM were reported; however, one particular analogue (compound **5**) was identified as being more effective, with a reported IC_50_ of less than 1 µM; compound **6** showed an IC_50_ in the range of 1–5 µM, and compound **2** in the range of 5–10 µM [63,64].

As mentioned above, the FASII pathway is essential for the de novo synthesis of short-chain fatty acids, such as myristic acid and palmitic acid [65]. These fatty acids are then used as building blocks for complex lipids, including phosphatidylcholine (PC) and other acylglycerol derivatives such as diacylglycerol (DG) [66,67]. Martins-Duarte et al. [68] report that TLM analogues affect acylglycerol synthesis, which is consistent with the inhibition of the FASII pathway, since blocking the production of precursors stops the synthesis of downstream structural lipids [68].

Transmission electron microscopy observations show that TLM analogues interfere with division and membrane organelles, which is directly related to their mechanism of action, since the FASII pathway, altered by TLM analogues, is essential for organelle biogenesis and parasite survival, as synthesized fatty acids are vital components of cell membranes and, therefore, organelles [43,63].

When fatty acid synthesis in the apicoplast is inhibited, the structure of the membranes is altered, ultimately resulting in the death of the parasites. Disruption of the apicoplast affects parasite division and proliferation. Alteration of lipid synthesis (including glycerophospholipids, which are part of cell membranes) affects cellular processes such as division, replication, and egress. The fact that TLM analogues interfere with division and organelles demonstrates that they are acting on the FASII pathway, resulting in dysfunction and growth inhibition [26].

### 6.10. FabD Inhibitors

In *Toxoplasma gondii*, the components of the FASII pathway have been recognized as potential drug targets; however, the enzyme malonyl CoA: ACP transacylase (FabD) has not yet been explored as a drug target. Therefore, Mamidi et al. (2015) [69] used a computational approach based on virtual screening and PLS (partial least squares) modelling to identify compounds that potentially target the enzyme malonyl-CoA: ACP transacylase (FabD) [69].

The authors used a dataset consisting of 45,138 compounds obtained from the ZINC database: ZINC drug database (Zdd), ZINC in man (Zim), and ZINC natural derivatives (Znd). Prior to analysis, the compounds were filtered based on certain physicochemical parameters, including molecular weight (32–350 g/mol), XlogP (−4 to 3.5), polar surface area (0–200 Å^2^), and number of rotatable bonds (1–7) [69].

The results showed that several compounds had a higher binding affinity than the natural substrate of FabD, malonyl thioester pantothenate; most of the compounds were derivatives of natural products, highlighting their potential as selective inhibitors of FabD in *T. gondii*. The evidence presented by the authors on the reported activities is in silico in nature, obtained through consensus docking calculations and molecular dynamics simulation [69,70].

#### Inhibitors Identified for TgFabD

PLS modelling identified imidazoles as the main chemical scaffold associated with specificity toward the TgFabD enzyme of *T. gondii* and with increased binding affinity [69]. Imidazole, a five-membered aromatic heterocycle containing two non-adjacent nitrogen atoms, stood out among the structural properties evaluated. In this analysis, several compounds approved for human use exhibited high affinity for TgFabD, including telbivudine (1-(2-deoxy-β-L-erythro-pentofuranosyl)-5-methylpyrimidine-2,4(1H,3H)-dione) and 2-ethyl-1,3-hexanediol, as well as other ligands with pharmacological potential such as 1,2-O-isopropylidene-D-xylofuranose, 1-(3-ethoxyphenyl)-2-methylaminoethanol, 3-(3,4-dimethoxyphenyl)propylamine, and 1-(methylamino)-3-phenoxypropan-2-ol, all of which exhibited relevant interactions with the enzyme’s active site through hydrogen bonds and Van der Waals forces [69,71].

The compounds mentioned have some structural differences that explain their ability to form non-covalent interactions with TgFabD. This demonstrates their potential as inhibitors, according to molecular modelling studies [69]. Telbivudine is a nucleoside analogue that is active against HBV and acts in its triphosphate form, and molecules such as 2-ethyl-1,3-hexanediol and 1,2-O-isopropylidene-D-xylofuranose are notable for their ability to form hydrogen bond aromatic derivatives, which also exhibit these properties, such as 1-(3-ethoxyphenyl)-2-methylaminoethanol and 3-(3,4-Dimethoxyphenylpropylamine), which have functional groups capable of interacting with various biological targets, reinforcing its usefulness in drug design studies [69].

The in-silico results reported show an inhibition mechanism based on the occupation and blocking of the catalytic interaction space of TgFabD. These use electrostatic energies, van der Waals forces, and binding free energy (ΔG) as some variables to predict inhibitory affinity and establish structure-function relationships [69].

### 6.11. Salicylanilides

Salicylanilides (considered salicylic acid analogues or substituted amides of salicylic acid) have been identified as a promising group of inhibitors against *T. gondii*. These compounds have demonstrated significant antiparasitic activity both in vitro and in vivo [46]. This study indicates that some salicylanilide derivatives at low nanomolar concentrations exhibit in vitro activity, demonstrating high potency and efficacy against different strains of the parasite. In particular, compounds **3i**, **3j**, **7a**, **14a**, and **14b** stood out for their efficacy against *T. gondii* tachyzoites (RH, RH-YFP, and ME49 strains). The authors of this study focused on the search for inhibitors with structural modifications in the triclosan scaffold to optimize their biological and pharmacological properties [46].

Compounds **14a** and **14b** were also eval1uated in vivo in a murine model (Swiss Webster) infected with the RH strain of *T. gondii*, corresponding to an acute infection. Both compounds are effective in reducing parasite load after oral administration of 100 mg/kg and 25 mg/kg, which increased the survival of infected mice [46]. The exact mechanism of action of salicylanilides is not yet fully determined, but it has been proposed that these compounds along with other substituted salicylic acid amides, act as inhibitors of the enoyl ACP reductase (FabI) enzyme of the FASII pathway [46].

### 6.12. Benzimidazole

Current studies on the enoyl-ACP reductase (ENR) enzyme of *T. gondii* have identified two main groups of inhibitors: classical NAD+ dependent ENR inhibitors and, more recently, a family based on benzimidazole derivatives that act on the NADH bound ENR enzyme [51,72].

Classic inhibitors such as isoniazid, diazaborines, and triclosan are mentioned primarily as historical references due to their demonstrated inhibitory activity, ease of synthesis, and presence in some pharmaceutical and household products. Triclosan is distinguished by its strong affinity for NNRs and its widespread use as an antimicrobial agent [40,72].

On the other hand, benzimidazole-based inhibitors form a more recent family of compounds of interest that were originally designed to act on the NADH-bound ENR enzyme, considered a pharmacological target due to its higher affinity compared to the NAD+ dependent form [73,74].

In *Toxoplasma gondii,* three compounds were evaluated: compound **1** (a 3,4-dichloro-substituted benzimidazole), compound **2**, and compound **3**. Compounds **2** and **3**, described by Wilkinson, are benzimidazole derivatives synthesized as analogues of the main compound (compound **1**). These compounds were obtained from commercially available substituted 5,6-dimethylbenzimidazole and benzyl bromides via a reaction with NaH and KI in DMF (dimethylformamide). The synthesis of these compounds has been reported to be successful, achieving a purity greater than 95%, which was determined by HPLC [75].

In initial studies, these compounds showed moderate inhibitory activity against *Francisella tularensis* (FtENR), where they reported an IC_50_ value of 300 nM for compound **1** [76]. The proposed mechanism of action for benzimidazole inhibitors is based on their ability to bind to the active site of NADH-dependent ENR. In the case of *F. tularensis*, compound **1** forms a hydrogen bond with the conserved catalytic tyrosine and the NADH cofactor, which stabilizes its binding to the active site [76]. Unlike triclosan, these inhibitors do not exhibit π–stacking interactions with the cofactor, which could explain differences in their efficacy [73,74].

However, in *T. gondii*, experimental results revealed low or even no enzymatic inhibition of TgENR by the compounds, even at concentrations of 1 μM, despite showing good antiparasitic activity in cell cultures. This discrepancy between low enzymatic potency (IC_50_) and high cellular efficacy (MIC_50_) suggests an off-target effect, i.e., an alternative mechanism of action not directly related to ENR inhibition [54]. A search for possible alternative targets in the PubChem database revealed that benzimidazole-like structural scaffolds can interact with various proteins, such as sterol 14α-demethylase, galanin receptor 3, pteridine reductase 1, TRPC4 cation channel, and aldosterone synthase. However, most of these targets are not present in Apicomplexa parasites, including *T. gondii,* which lacks sterol 14α-demethylase and depends on cholesterol sequestration from the host; therefore, the specific molecular target responsible for the antiparasitic activity of benzimidazoles observed in *T. gondii* has not yet been identified [54].

Benzimidazole compounds showed promising in vitro activity with MIC_50_ values between 1 and 10 μM against type I (RH) and type II (Pru) strains of *T. gondii*. Specifically, compound **1** had an MIC_50_ of 2.5 μM, while compound **2** had an MIC_50_ of 4 μM against the RH strain. Furthermore, compounds **1** and **3** significantly reduced the growth of the Pru strain at concentrations of 10 μM, confirming their potential as antiparasitic agents [75]. Liauw et al. (2014) [72] attempted to co-crystallize TgENR with compound **1** using high concentrations of the inhibitor (3.2 mM); however, this showed no evidence of binding in the electron density map. This suggests relevant structural differences between TgENR and FtENR, particularly in the Ser242–Asp249 loop region, and the substitution of methionine for alanine, which reduces the inhibitor’s ability to stabilize the active site, explaining the loss of affinity of benzimidazoles for the *T. gondii* enzyme [75].

The authors also showed that the compounds exhibit low cellular toxicity against human fibroblasts (HFF) and PC3-Luc cells, even at concentrations of 10 μM, suggesting a wide therapeutic margin. The combination of good antiparasitic activity and low toxicity makes benzimidazole derivatives a promising family for the development of new inhibitors targeting *T. gondii,* despite their limited action on TgENR [75]. While current data support the in vitro efficacy of benzimidazole inhibitors, there are no reports of in vivo studies confirming their activity or determining their exact molecular target (Table 2) [75]. 

## 7. Therapeutic Perspectives

The development of FASII pathway inhibitors remains a crucial therapeutic strategy against Apicomplexa parasites such as *T. gondii*. This pathway is an attractive target because it differs from the FASI pathway found in mammals [26,77].

Historically, disruption of FASII, either genetically (such as removal of the acyl carrier protein, ACP) or pharmacologically (triclosan), has been shown to be essential for parasite survival and pathogenesis in vitro and in vivo [26]. Major advances have focused on optimizing triclosan for its potent ability to inhibit the FASII enzyme enoyl-ACP reductase (FabI). Since triclosan has poor pharmacokinetic properties, such as low solubility and permeability, medicinal chemistry efforts have focused on designing triclosan analogues with improved ADMET (absorption, distribution, metabolism, excretion, and toxicity) profiles [41,50,54].

In addition, alternative enzymes in the FASII pathway, such as malonyl-CoA-ACP transacylase (FabD) and β-hydroxyacyl-ACP dehydratase (FabZ), have been explored. In a recent experimental study, the elimination of FabZ in *T. gondii* through gene editing led to a significant reduction in vitro growth, partial loss of the apicoplast, and decreased virulence in murine models, confirming that FabZ is crucial for the survival of the parasite [36]. (Table 2).

Although *T. gondii* could capture and remodel host lipids, these compensatory mechanisms are not sufficient to meet its high lipid demands during intracellular replication [78]. *T. gondii* incorporates fatty acids, phospholipids, and cholesterol from host cell membranes via transporters and contact zones between the host endoplasmic reticulum and the parasitophorous vacuole [79].

It also has the ability to elongate and desaturate its exogenous lipids to adapt them to its needs. However, these salvage pathways only complement, but do not replace, de novo synthesis mediated by the FASII pathway. Since this pathway has been reported to be essential for maintaining the parasite’s virulence [12], the development of FASII inhibitors requires compounds with pharmacokinetic properties capable of crossing biological barriers (such as the blood–brain barrier) to better reach the apicoplast. Liposomal nanoparticle formulations, as demonstrated with triclosan, have been shown to improve efficacy by reducing the required dose and minimizing toxicity, representing a promising approach for targeted therapies against *T. gondii*.

## 8. Conclusions

The evidence analyzed highlights that the FASII pathway, located in the apicoplast, is one of the most promising and selective therapeutic targets against toxoplasmosis due to its absence in mammalian cells and its potential to offer greater specificity than conventional treatments. In this context, the identification of new targets within this pathway, along with the use of computational tools for rational drug design, has significantly broadened the therapeutic landscape. Therefore, future research should focus on developing compounds that combine high molecular specificity with suitable pharmacological properties capable of ensuring their bioavailability and access to tissues such as the central nervous system.

## Figures and Tables

**Table 1:** Differences between FASI and FASII synthesis pathways.

Characteristic	FASI	FASII	Reference
Structure	Multifunctional unique polypeptide complex	Multiple monofunctional enzymes.	[26,29]
Substrate transfer	Intermediates are transferred between domains within the same polypeptide.	Intermediates are transferred between enzymes via acyl carrier protein (ACP).	[26,29]
Enzymes	A single multifunctional protein complex.	A series of monofunctional enzymes (e.g., ketoacyl synthase, dehydratase, reductase).	[26,29]
Location	In cytoplasm of eukaryotes (such as humans), fungi, and some bacteria (such as Corynebacterium, Mycobacterium, and Nocardia).	Bacteria, archaea, plants, animal mitochondria, and the apicoplasts of some Apicomplexa parasites (e.g., in *T. gondii*)	[26,29]
Function	Mainly responsible for the de novo synthesis of fatty acids in eukaryotic cells.	In Apicomplexa, it is responsible for the de novo production of fatty acids (C14:0, C16:0) that are not provided by the host and for specific elongation pathways.	[26,29]
Pharmacological application	Generally, not a good target for drugs in humans, as it would cause significant side effects.	It is a potential therapeutic target in bacteria and Apicomplexa because it is essential and not present in humans.	[26,29]

Table compiled from information reported by [26] and [29].

**Table 2:** Table of inhibitors and their target enzymes.

Inhibitor	Enzyme/Molecular Target	Mechanism of Action	Reference
Pyrimethamine	Dihydrofolate reductase (DHFR)	Inhibits the parasite’s DHFR, interfering with the synthesis of nucleotides and certain amino acids (first-line treatment).	[12]
Sulfadiazine	Dihydropterate synthase (DHPS)	It acts as a competitive analogue of aminobenzoic acid, an essential substrate for this enzyme (first-line treatment).	[13]
Macrolides (Azithromycin, Spiramycin) and Lincosamides (Clindamycin)	50S ribosomal subunit	They interrupt protein synthesis in the parasite’s apicoplast.	[14,15]
Triclosan	Enoil-Acyl Carrier Protein Reductase (ENR or FabI) (specifically TgENR)	It was one of the first compounds identified to act on bacterial and apicomplexan FASII. It inhibits TgENR activity by forming a tight ternary complex with the enzyme and the oxidized cofactor (NAD+).	[40,41]
Thiolactomycin (TLM)	β-ketoacyl-ACP synthase (FabH)	It specifically targets bacterial type II fatty acid synthesis and has demonstrated activity against *T. gondii*. It catalyzes a step in the FASII pathway.	[61,63]
Tiolactomycin (TLM) analogues	FASII pathway	Designed to optimize pharmacological properties, they target the FASII pathway, interfering with the synthesis of acylglycerol and structural lipids.	[61,63]
Triclosan analogues (e.g., triazole analogues (**16c**, **16a**, **16b**), **41b**, **37a**, **37c**, **24a**, **24b**, **33**, **5c**, **14b**, **18**, and potent compounds **5**, **9**, **10**, **15**)	Enoil-Acyl Carrier Protein Reductase (TgENR or FabI)	Developed to improve the ADMET properties of triclosan. All potent analogues share the same mechanism of action, binding to the TgENR/NAD+ complex.	[50,54]
Salicylanilides (e.g., **3i**, **3j**, **7a**, **14a**, **14b**)	Enoil-Acyl Carrier Protein Reductase (ENR/FabI) (Proposed mechanism)	Identified as promising inhibitors with high antiparasitic activity. Their exact mechanism of action is not fully determined, but it is proposed that they inhibit ENR/FabI of FASII.	[46]
Benzimidazoles (Compounds **1**, **2**, **3**)	Enoil-Acyl Carrier Protein Reductase (NADH-dependent ENR) (Original/designed target); Extradiane target (off-target)	They were designed to target NADH-dependent ENR. However, they showed low or no enzymatic inhibition of TgENR in *T. gondii*, suggesting an alternative mechanism of action unrelated to ENR. The specific molecular target has not yet been identified.	[46]
Compounds identified in silico (e.g., telbivudine, 2-ethyl-1,3-hexanediol, 1,2-O-isopropylidene-D-xylofuranose, 1-(3-ethoxy-phenyl)-2-methylamino-ethanol, etc.).	Malonyl-CoA: ACP transacylase (FabD)	Identified through virtual screening and PLS modelling as compounds potentially targeting the FabD enzyme, demonstrating binding affinity through in silico calculations.	[69]

## Data Availability

Data are contained within the article.

## Abbreviations

The following abbreviations are used in this manuscript:

P/S	Pyrimethamine and sulfadiazine
FAS I	Type I fatty acid synthesis
FAS II	Type II fatty acid synthesis
ELO	Elongation pathway
ACP	Acyl carrier protein
PDH	Pyruvate dehydrogenase
ENR	Enoyl-ACP reductase
ACC	Acetyl CoA carboxylase
Fab D	Malonyl CoA-ACP
Fab H	β-ketoacyl-ACP synthase III
Fab G	3-oxoacyl-ACP-reductase
Fab Z	β-hydroxyacil-ACP dehydratase
Fab I	Enoil-ACP-reductase
TgAT1	Acetyl-CoA transporter
nM	Nanomolar
IC_50_	Median inhibitory concentration
MIC_50_	Minimum inhibitory concentration
ADMET	Absorption, distribution, metabolism, excretion, and toxicity
Sw	Water solubility
Kd	Dissociation constant
TSA	Thermal displacement assay
TLM	Thiolactomycin
PLS	Partial least squares
DMF	Dimethylformamide
DHFR	Dihydrofolate reductase
DHPS	Dihydropterate synthase
